# Open and Arthroscopic Surgical Treatment of Femoroacetabular Impingement

**DOI:** 10.3389/fsurg.2015.00063

**Published:** 2015-12-02

**Authors:** Benjamin D. Kuhns, Rachel M. Frank, Luis Pulido

**Affiliations:** ^1^Hip Preservation Center, Division of Sports Medicine, Department of Orthopedic Surgery, Rush University Medical Center, Chicago, IL, USA; ^2^Houston Methodist Orthopedics & Sports Medicine, Houston, TX, USA

**Keywords:** femoroacetabular impingement, hip arthroscopy, open surgical dislocation, surgical techniques, sports medicine, periacetabular osteotomy

## Abstract

Femoroacetabular impingement (FAI) is a common cause of hip pain, and when indicated, can be successfully managed through open surgery or hip arthroscopy. The goal of this review is to describe the different approaches to the surgical treatment of FAI. We present the indications, surgical technique, rehabilitation, and complications associated with (1) open hip dislocation, (2) reverse periacetabular osteotomy, (3) the direct anterior “mini-open” approach, and (4) arthroscopic surgery for FAI.

## Introduction

Femoroacetabular impingement (FAI) is a common cause of hip pain and has been correlated to the development of arthritic changes in the young adult. FAI is a dynamic condition in which deformities in the acetabulum and/or femoral head–neck junction limit hip range of motion and generate abnormal intra-articular contact areas, causing early acetabular labrum and articular cartilage damage (1–3). Often affecting a young and active population, FAI presents with hip and groin pain as well as decreased range of motion. Three mechanisms of FAI have been classically described; cam, pincer, and combined. Cam is the femoral head asphericity and malformed femoral head–neck junction with decreased offset. Cam lesions are more frequently seen in males. The acetabular injury pattern includes labral damage and cartilage delamination through shear forces at the abutment between the abnormal femoral head–neck junction “cam” and the acetabular rim (2, 4). Pincer deformities, result from excessive acetabular coverage secondary to deep sockets (coxa profunda and protrusio), increased anterior acetabular coverage and true acetabular retroversion. A pincer impingement compresses the labrum between the acetabular overcoverage and the femoral neck with hip range of motion (3, 5). Combined deformities are a combination of these two mechanisms and are the most common variant of FAI (6, 7).

There are additional extra- and intra-articular anatomical conditions that must be recognized in FAI. Although combined deformities (cam and pincer) are the most common mechanism of FAI, acetabular dysplasia can coexist with cam impingement and must be considered in the surgical plan to avoid worsening of hip instability and early catastrophic failures. Femoral deformities include excessive femoral retrotorsion which promotes anterior impingement and coxa valga with excessive femoral antetorsion which leads to posterior impingement (8). Additionally, coxa vara may cause intra- and extra-articular impingement (9). The degree of pelvic tilt can also impact impingement pathology. Anterior pelvic tilt increases acetabular retroversion and results in early occurrence of FAI. If flexible, this dynamic conflict should improve with non-surgical treatment (10).

The degree of intra-articular deformity in patients with FAI is variable; however, it is the repetitive and extreme ranges of motion commonly seen in the athletic population that exacerbate the impingement and injury pattern to the labrum and articular cartilage. The effects of FAI can be devastating for highly active patients, often requiring activity modification and/or cessation. While often initially managed non-operatively, surgery is indicated for certain FAI patients to correct the osseous deformities and manage the associated chondrolabral lesions. The goal of surgical treatment include pain relief, improved function and range of motion, and possibly delay early onset of osteoarthritis (3, 7).

The aim of this review is to discuss different approaches for the surgical treatment of FAI, including open surgical hip dislocation (SHD), reverse periacetabular osteotomy (PAO), mini-open direct anterior approach, and hip arthroscopy. The decision to proceed with open versus arthroscopic surgery for surgical treatment of FAI should be based on the patient’s pathoanatomy, taking into account the surgeon’s experience and preference. In experienced hands, both open and arthroscopic treatments of FAI have shown good mid-term and long-term clinical results.

## Surgical Hip Dislocation for Treatment of FAI

The Ganz technique of SHD was described as a safe surgical approach to the femoral head and the acetabulum without the risk of avascular necrosis (11). Their observations allowed to refine the concept of FAI as a mechanical cause of hip osteoarthritis. SHD was the first described method of treatment, with satisfactory clinical results published at 5 and 10 years (12, 13).

Surgical hip dislocation is a successful treatment modality for most cases of FAI with the majority of patients returning to sports activities (14). The indications for open versus arthroscopic treatment of FAI are based on surgeon’s preference, skills, and experience. A recent systematic review including 16 studies and 600 patients comparing open versus arthroscopic treatment of FAI (level 4 evidence) showed that both approaches had similar clinical results when conversion to total hip arthroplasty was used as primary endpoint (15). In this review, hip arthroscopy was associated with higher postoperative general health-related quality of life scores 12-Item Short-Form Survey (SF-12).

Surgical hip dislocation is the preferred surgical technique for patients with FAI and a high-riding trochanter from old Perthes or slipped capital femoral epiphysis. The main advantage is the possibility of performing a trochanteric advancement and relative neck lengthening, to optimize abductors biomechanics and correct associated extra-articular impingement.

Surgical hip dislocation can also be a better alternative in certain clinical scenarios that are difficult to address with hip arthroscopy, including:

1. Anticipated labral reconstruction (fascia lata or round ligament autograft).
2. Coxa profunda or global overcoverage.
3. Posterolateral (PL) cam lesions extending over the retinacular vessels.

### Contraindications for SHD to Treat FAI

1. Patients 40 years old and older (16).
2. Extensive cartilage damage.
3. Anterior hip subluxation.
4. Anterior and posterior cartilage damage (coup–countercoup).
5. Smokers.

### Surgical Hip Dislocation Technique

The technique as described by Ganz et al. is basically an anterior dislocation of the hip after a trochanteric flip osteotomy, avoiding injury to medial femoral circumflex artery (MFCA) maintaining the blood supply to the femoral head (11). The patient is placed on the lateral decubitus position, and a 15 cm straight lateral incision centered over the greater trochanter is performed for a Gibson approach to the hip. The trochanteric trigastric (flip) osteotomy is performed starting 5 mm anterior (lateral) to the greater trochanter overhang, cutting with a small oscillating saw from the posterior greater trochanter toward the vastus ridge and anterior greater trochanter. After completing the osteotomy, the gluteus medius tendon, the long tendon of the gluteus minimus, and the vastus lateralis remain attached to the mobile greater trochanter fragment.

The proximal vastus lateralis is elevated to free up the mobile trochanteric fragment. Flexion, abduction, and external rotation of the hip releases tension of the flip osteotomy and facilitates the exposure of the hip capsule. Proximally visualize the attachment of the piriformis tendon into the stable greater trochanter and identify the interval between the piriformis and remnants of gluteus minimus. The hip capsule is approached through this interval by sharp dissection of the gluteus minimus from the capsule. This interval is safe with regard to vascularity of the femoral head as the anastomosis between the deep branch of the MFCA and inferior gluteal artery runs inferior to the piriformis (17).

**Figure 1:**
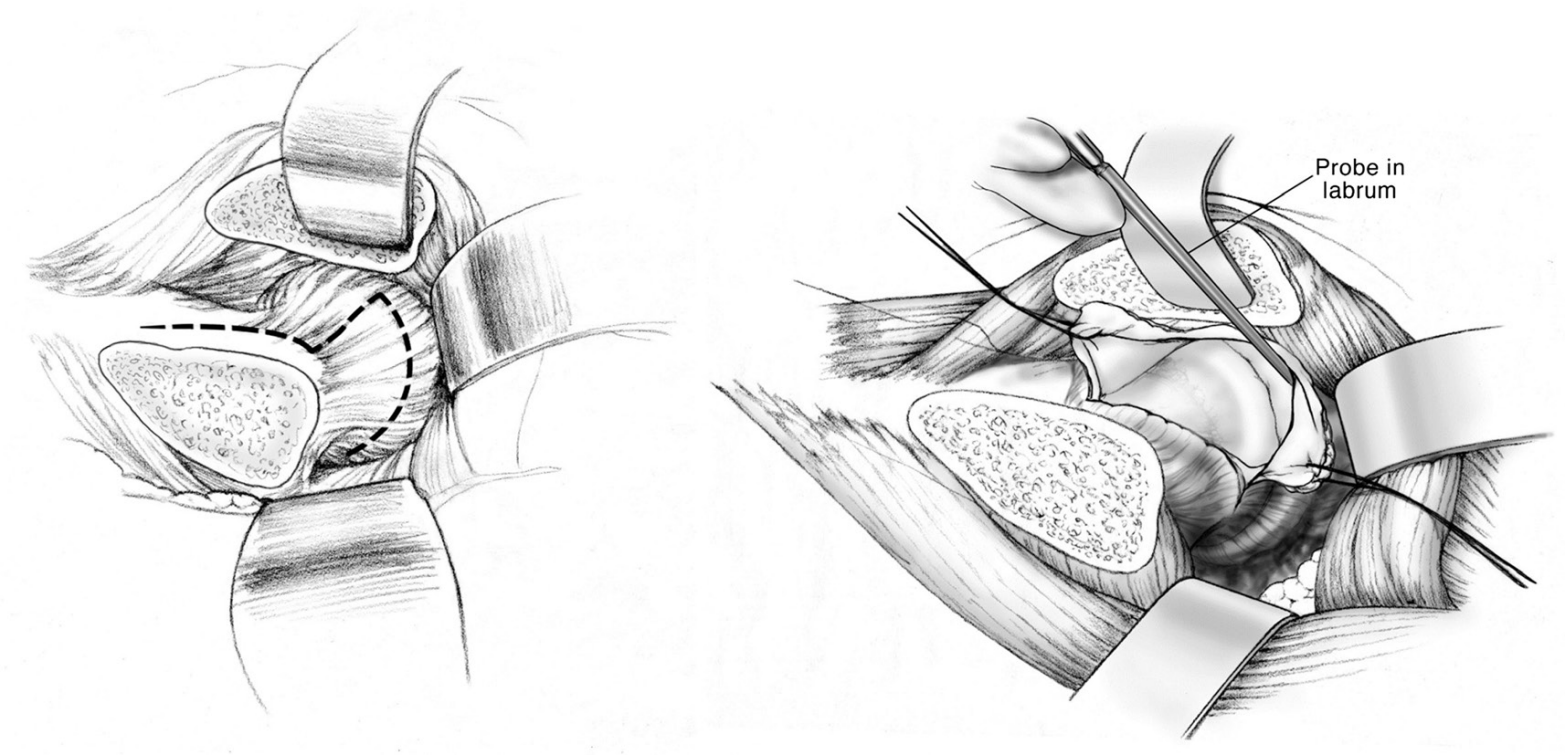
**Anterior Z capsulotomy**. Adapted with permission from Dr. Rafael J. Sierra and the Mayo Foundation, Rochester, MN, USA.

The capsulotomy is performed starting at the anterosuperior edge of the stable trochanter toward the acetabular rim along the long axis of the neck from distal to proximal. The distal and anterior transverse limb of the capsulotomy is performed and tagged, followed by the proximal and posterior limb of the capsulotomy, performing a Z-shaped capsulotomy for the right hip, and an inverse Z-shaped capsulotomy for the left hip (Figure 1). At this point, the peripheral compartment and acetabular rim of the hip joint is exposed, and direct assessment of FAI with range of motion is performed. With a hook around the inferior femoral neck, the hip is externally rotated and subluxated, allowing to cut the round ligament with angled scissors. The femoral head can then be dislocated and pushed posteriorly by abduction, flexion, and external rotation allowing a 360° visualization of the acetabulum. At this point, there is full access to the acetabular rim allowing inspection and management of the labrum and acetabular cartilage (17).

**Figure 2:**
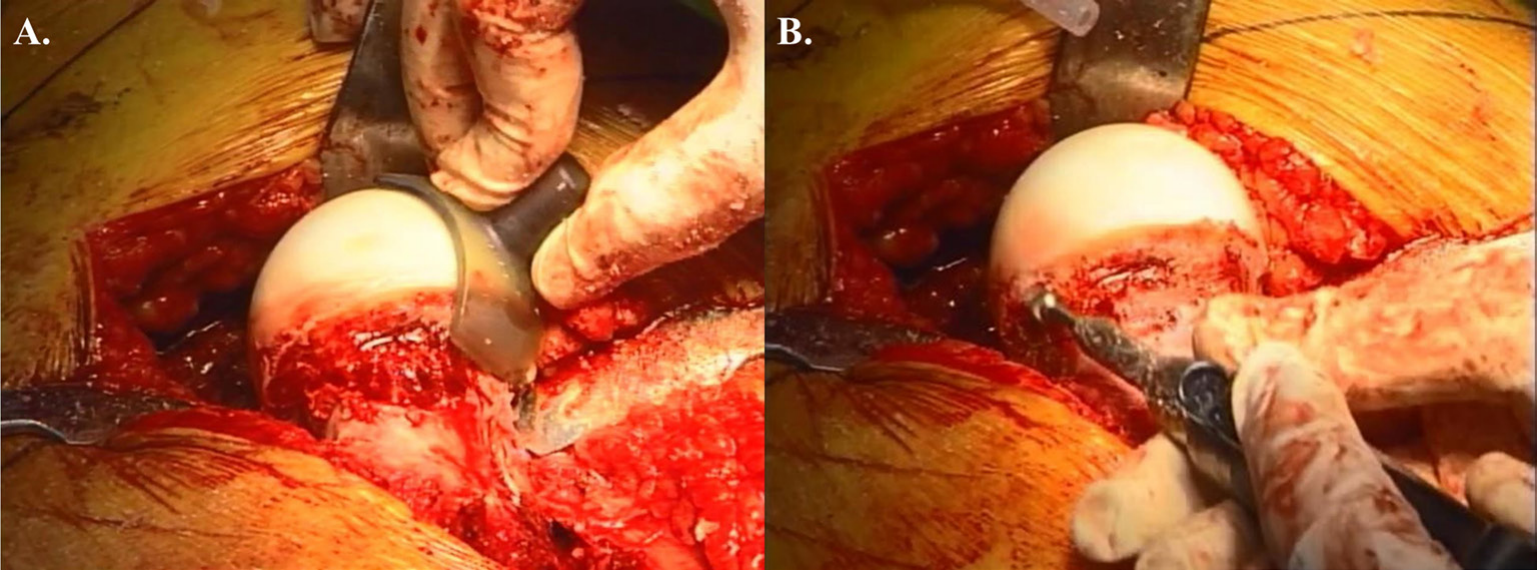
**Open treatment of cam lesion**. **(A)** Restoration of femoral head–neck offset and head sphericity with intraoperative templates. **(B)** The high speed burr can be used safely in the posterolateral area with direct visualization of the retinacular vessels. Adapted with permission from Dr. Robert T. Trousdale and the Mayo Foundation, Rochester, MN, USA.

The hip is then adducted for evaluation and management of abnormalities at the femoral head and neck. Hemispherical plastic templates are used to guide bone resection using a high speed burr or osteotomes to restore head sphericity and head–neck offset (Figure 2). The hip is then reduced and tested for range of motion and impingement. The tagged ends of the capsule are approximated with interrupted sutures avoiding tension that may adversely affect the perfusion to the femoral head. The mobile trochanteric fragment is reduced in anatomic position and fixed with two 4.5 mm screws aiming toward the lesser trochanter. Layered closure including the fascia, subcutaneous tissues, and skin is then performed.

An extended retinacular soft tissue flap can be performed during SHD in cases of proximal femoral deformities with a high-riding trochanter. This technique allows performing relative neck lengthening and management of intra- and extra-articular impingement, improving pain, ROM, and abductors strength (18).

### Rehabilitation After Surgical Hip Dislocation

Patients are mobilized the day following surgery. Passive- and active-assisted internal or external rotation is permitted to protect trochanteric fixation. Passive ROM is initiated immediately with the use of a continuous passive motion (CPM) machine 6 h a day for 6 weeks to decrease the risk of hip joint adhesions. A stationary bike may begin at week 2. The patients are touch weight bearing for the first 4 weeks, and weight bearing is advanced after 4 weeks. Hip flexion is limited to 90°. Muscle weakness may persist for 3 months after surgery, and abductor rehabilitation is continued throughout the ensuing months. The patients are seen at 8 weeks after surgery, and at that time patients are typically using one crutch or no support. A physical therapist supervises the return to high impact pivoting sports, which usually does not occur before 6 months.

### Complications Associated with Surgical Hip Dislocation

Surgical hip dislocation is a safe procedure with low reported complication rates. Potential complications include osteonecrosis, femoral neck fracture, trochanteric non-union, nerve injury, heterotopic ossification (HO), and thromboembolic disease. Sink et al. published a multicenter study looking at the complications after 334 SHDs in 302 patients. There were no cases of osteonecrosis or femoral neck fracture in their series. They reported one case of temporary sciatic nerve injury that resolved. Trochanteric non-union was the most serious complication with a prevalence rate of 1.8% (stx cases of 334). There were two cases of deep vein thrombosis and one deep infection. The most common complication was mild HO that did not require treatment (19).

## Reverse Periacetabular Osteotomy for True Acetabular Retroversion

**Figure 3:**
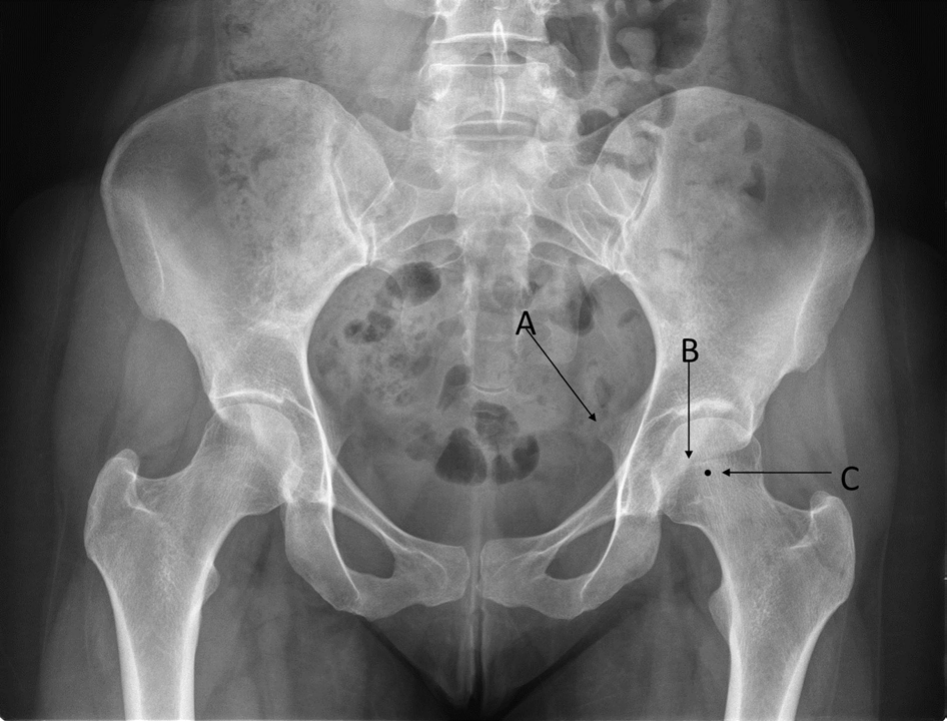
**The radiographic findings of acetabular retroversion consist of a positive ischial spine sign (A), crossover sign (B), and a posterior wall sign (C)**. The small acetabular size and volume represents a challenge for surgical treatment. Adapted with permission from Dr. Rafael J. Sierra and the Mayo Foundation, Rochester, MN, USA.

True acetabular retroversion is secondary to an external rotation deformity of the affected hemipelvis and is a known cause of pincer FAI (20). The radiographic findings on a true AP pelvis consist of a positive crossover sign, posterior wall sign, and ischial spine sign (Figure 3). FAI secondary to acetabular retroversion is successfully treated with a reverse PAO, which corrects the underlying deformity, improving hip pain, and range of motion (20, 21). Combined FAI consisting in true acetabular retroversion and associated cam lesions can be treated with a reverse PAO. For these cases, the incision is extended distally for an anterior hip capsulotomy, for femoral head–neck osteochondroplasty, and for management of the labrum pathology (20).

The acetabular correction during reverse PAO is a challenging step, and specially attention to avoid increasing the lateral edge angle or ending with a negative Tonnis angle during the anteversion maneuver of the mobile fragment is critical. Overcorrection of the acetabular fragment must be avoided to prevent excessive acetabular anteversion, posterior acetabular impingement, and poor clinical results (21).

### Periacetabular Osteotomy Technique

**Figure 4:**
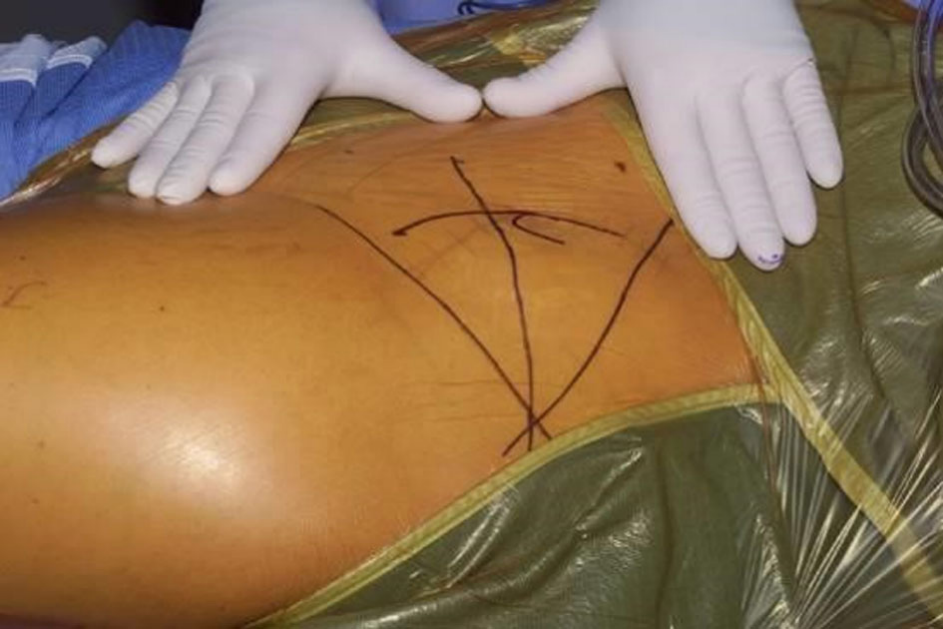
**Longitudinal c-shaped 10–12 cm incision centered over the anterior superior iliac spine for a modified Smith-Petersen approach**. Adapted with permission from Dr. Robert T. Trousdale and the Mayo Foundation, Rochester, MN, USA.

**Figure 5:**
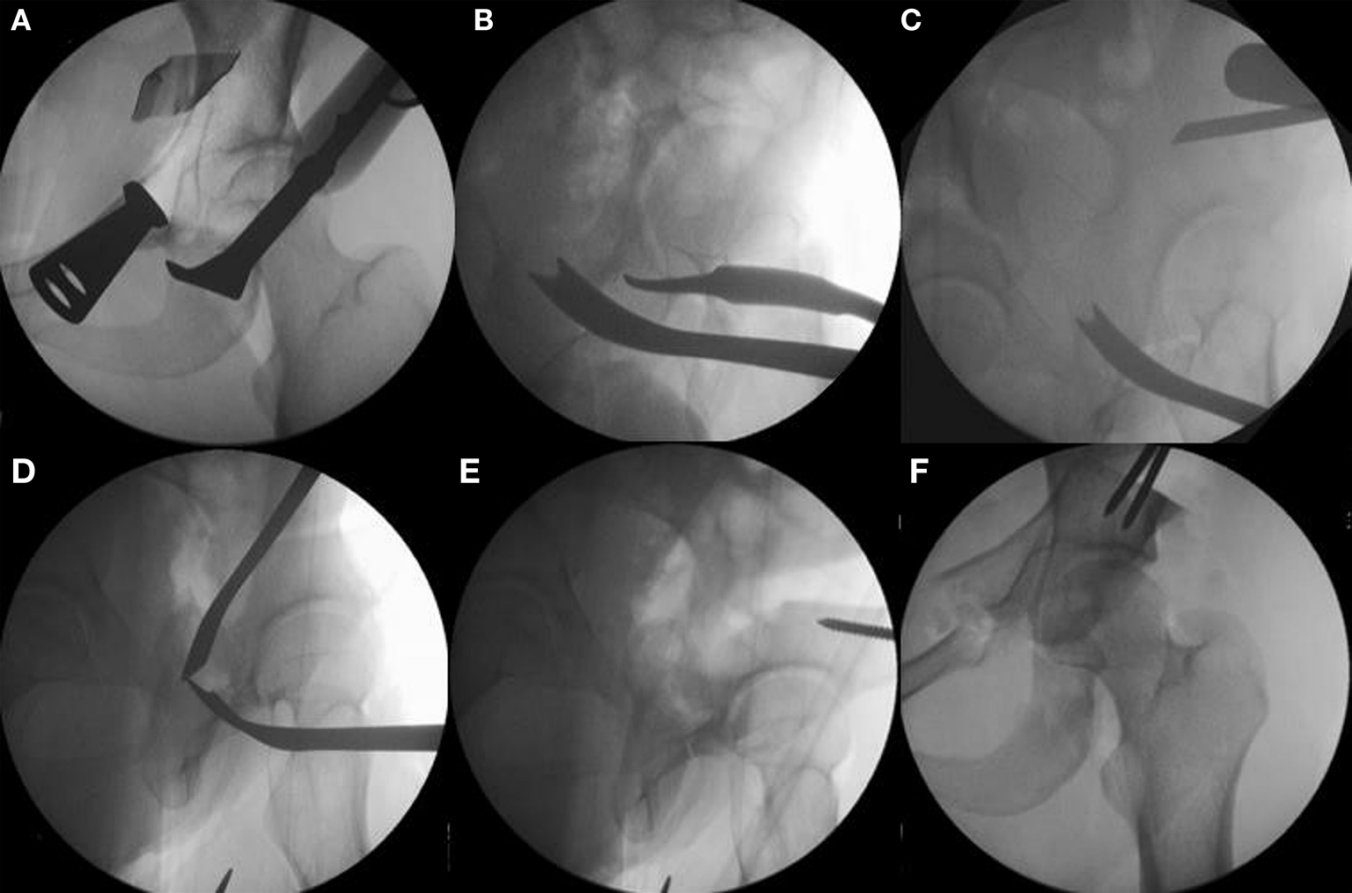
**The sequence of periacetabular osteotomies is performed under fluoroscopic guidance**. The first bone cut is the ischial osteotomy which is an incomplete osteotomy of 2.0–2.5 cm using a curved “Ganz” osteotome just inferior to the acetabulum [**(A)** the anterior to posterior fluoroscopic view and **(B)** a 65° lateral “false profile” view]. The second osteotomy is done at the superior pubic rami with osteotomes or a small saw. The third is the transverse iliac osteotomy **(C)**. Mark with fluoroscopy starting just distal to the anterior superior iliac spine aiming slight proximal, stop 1 cm lateral of the pelvic brim. The fourth and last osteotomy is the retroacetabular osteotomy that connects the first ischial with the third transverse iliac osteotomies. Use fluoroscopy (65° “false profile” view) to facilitate this osteotomy and avoid exiting into the hip joint or posterior column **(D)**. Once complete, the fragment should be mobile and ready for acetabular correction. Use a Schanz pin in the mobile fragment to facilitate control and mobilization of acetabulum **(E)**. Correct the anteversion of the mobile fragment, pin it, and check the hip range of motion **(F)**. Adapted with permission from Dr. Robert T. Trousdale and the Mayo Foundation, Rochester, MN, USA.

The surgical approach and osteotomies performed for an anteverting PAO (reverse PAO) for acetabular retroversion are performed in a similar manner and sequence as described originally for the treatment of hip dysplasia (22–24). The operation is done with the patient in the supine position through a modified Smith-Petersen approach using a longitudinal c-shaped (10–12 cm) incision centered over the anterosuperior iliac spine (Figure 4). The interval between tensor and sartorius is created, followed by subperiosteal elevation of the sartorius and the abdominal oblique muscles from the iliac crest. The subperiosteal elevation is continued down the inner pelvis toward the pelvic rim, and the anterior inferior iliac spine and the origin of the rectus femoris muscle are identified. The tendon of rectus femoris origin can be preserved or transected, tagged, and repaired without affecting the ability to reorient the acetabulum or affecting the clinical outcome (25, 26) The approach is then continued medial to the rectus retracting the iliopsoas tendon medially for exposure of the medial hip capsule, ischium, and the superior pubic ramus. Hip flexion decreases the tension over the iliopsoas and facilitates placement of a medial retractor over the superior pubic rami. A sequence of osteotomies is then performed using fluoroscopic guidance (Figure 5). The interval between the inferomedial capsule and iliopsoas is bluntly developed to allow placement of the angled osteotome for the first ischial osteotomy, which is an incomplete osteotomy of 2.0–2.5 cm just inferior to the acetabulum curving toward the posterior acetabular column (24). The second bone cut is an extra-articular and complete osteotomy of the superior pubic rami using osteotomes or a small oscillating saw. The third bone cut is the transverse iliac osteotomy starting just distal to the anterior superior iliac spine (ASIS) aiming slight proximal and stopping approximately 1 cm lateral of the pelvic brim. The fourth and last osteotomy is the retroacetabular osteotomy that connects the first ischial with the third transverse iliac osteotomies. This osteotomy is facilitated with the use of fluoroscopy (false profile view) to avoid exiting into the hip joint or posterior column. Once complete, the acetabular fragment should be mobile and ready for correction. The use of a Schanz pin in the mobile acetabular fragment helps to control it and to mobilize it. The acetabular reorientation into the correct amount of anteversion is performed and temporarily pinned. If the hip range of motion to impingement and fluoroscopic correction looks satisfactory, the acetabular fragment is fixed with three 4.5 screws from the stable pelvis into the fragment for definitive fixation (24).

## Direct Anterior Approach for Open Treatment of Anterior FAI

The direct anterior approach for open treatment of FAI was initially described by Ribas et al. (27). Their rationale was that FAI is a mechanical conflict of the anterior hip joint in the majority of cases. As such, it could be safely addressed with open surgery through a direct anterior approach without disrupting the trochanter and abductor mechanism (28).

The direct anterior approach uses a 4–12 cm longitudinal surgical incision starting 2 cm lateral and 2 cm distal to the ASIS aiming to the fibular head over the tensor muscle. Dissect down to fascia and incise the tensors fascia. Elevate the perimysium of the tensor muscle and access the anterior joint capsule trough the interval between the Sartorius and the tensor muscle. Ligate the branches of the lateral femoral circumflex artery and excise the pericapsular fat to access the anterior hip capsule. The capsulotomy is an I-shaped incision over the axis of the femoral neck. Manual traction can be performed to help visualize the central compartment through this approach. The femoral head and neck junction is visualized and the osteochondroplasty of the femoral neck is performed. Access to the labrum is limited to the anterior the acetabular rim.

Postoperatively, the patients are partial weight bearing using crutches for 6 weeks and then allowed full weight bearing. Physical therapy starts 6 weeks after surgery and return to full activity is typically 4–6 months postoperatively. Cohen et al. published a series of athletes with FAI treated with a direct anterior approach (28). They presented satisfactory clinical results with improved postoperative pain and activity levels. However, only 24 of 44 patients (55%) reported a return to their specific preoperative sports at an average follow-up of 22 months. The reported complications included hypoesthesia of the lateral femoral cutaneous nerve (LFCN) (20%) and one temporary femoral nerve palsy.

This technique has no added benefits from hip arthroscopy in the management of FAI. The disadvantages are mainly the limited visualization of the central compartment and the inability to address posterior and PL hip pathology. A single stage combined hip arthroscopy and anterior open cam resection has been described (29). However, the current advances in hip arthroscopy techniques do not justify this approach as cam deformity can be successfully managed at the time of hip arthroscopy.

## Arthroscopic Surgery for FAI

Advances in surgical techniques as well as in our understanding of the anatomic and biomechanical understanding of the native hip joint have prompted a dramatic increase in the arthroscopic management of FAI. While hip arthroscopy had previously been used to address chondral and labral tears, Philippon et al. were the first to report the technique to correct the osseous deformities present in FAI (30). Since 2006, arthroscopic management for FAI has increased over 600%, and one recent systematic review cited arthroscopy for FAI as the “preferred technique,” representing 50% surgical approaches compared to 34% for open surgical dislocation and 16% with the mini-open approach (31, 32). Additional improvements in instrumentation, visualization, and capsular management have expanded surgical indications to include a variety of intra-articular hip pathology coincident with FAI (33, 34).

### Set-Up and Portal Placement

**Figure 6:**
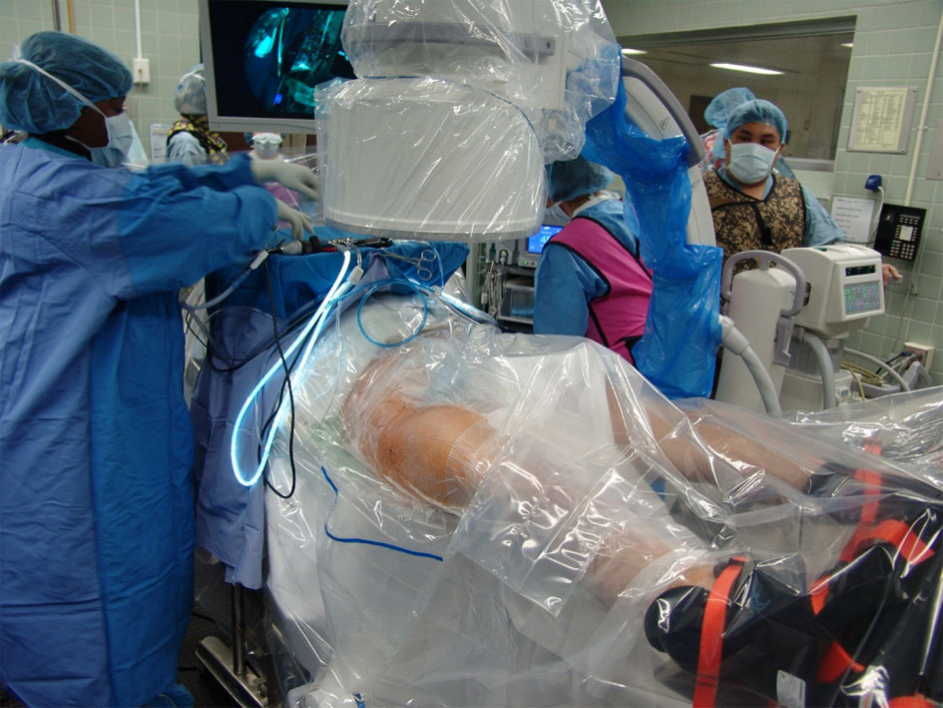
**Surgical set-up for right hip arthroscopy**. The patient is placed in the supine position on a traction table with attached hip distraction system (Smith and Nephew) and well-padded perineal post. Sterile draping is subsequently performed.

Hip arthroscopy can be performed on a standard fracture table or specialized traction table, in either the supine or the lateral position depending on surgeon preference (Figure 6) (35). Supine positioning is generally preferred, with a recent study reporting 100% of high volume hip arthroscopists preferring the supine over the lateral position (36). However, the lateral position may be favored in obese patients with a large pannus or in patients with large anterior osteophytes as these can hinder visualization in the supine position (37). Distraction is critical to gain access to the hip joint and work in the central compartment, and several distraction systems exist for both supine and lateral positions (35, 37, 38). Traction is typically achieved with the hip in abduction and internal rotation. Slight flexion (≤20°) during distraction is employed by some surgeons in order to relax the anterior capsule, which facilitates distraction and limits risk to femoral and sciatic nerve injury (38–40). A well-padded perineal post is commonly used to supply counter-traction and is padded to prevent postoperative pudendal neuropraxia. Once the hip is distracted, the central compartment can be accessed, initially by the introduction of a fluoroscopically guided spinal needle into the hip capsule. Typically, this occurs at the location of the anterolateral (AL) portal, anterosuperior to the proximal margin of the greater trochanter (35, 39). Penetration of the capsule produces a “vacuum sign” resulting from the equalization of intra-articular and atmospheric pressures (41). Distraction is critical to prevent iatrogenic chondral and labral injury when accessing the hip joint, with a recommended traction distance of at least 10 mm (40).

**Figure 7:**
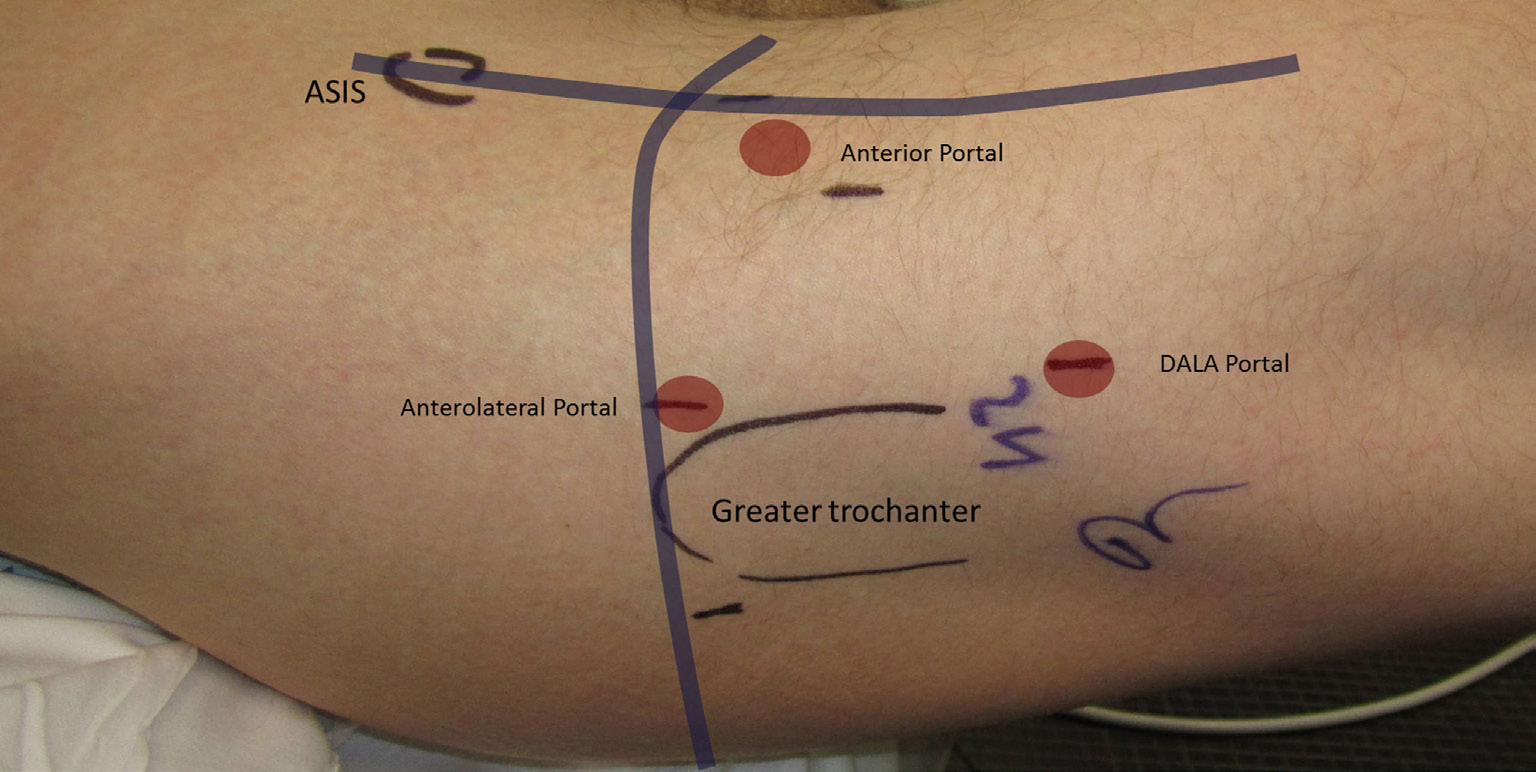
**Standard arthroscopic portal placement including the anterolateral, anterior, and distal anterolateral accessory (DALA) portals**.

**Figure 8:**
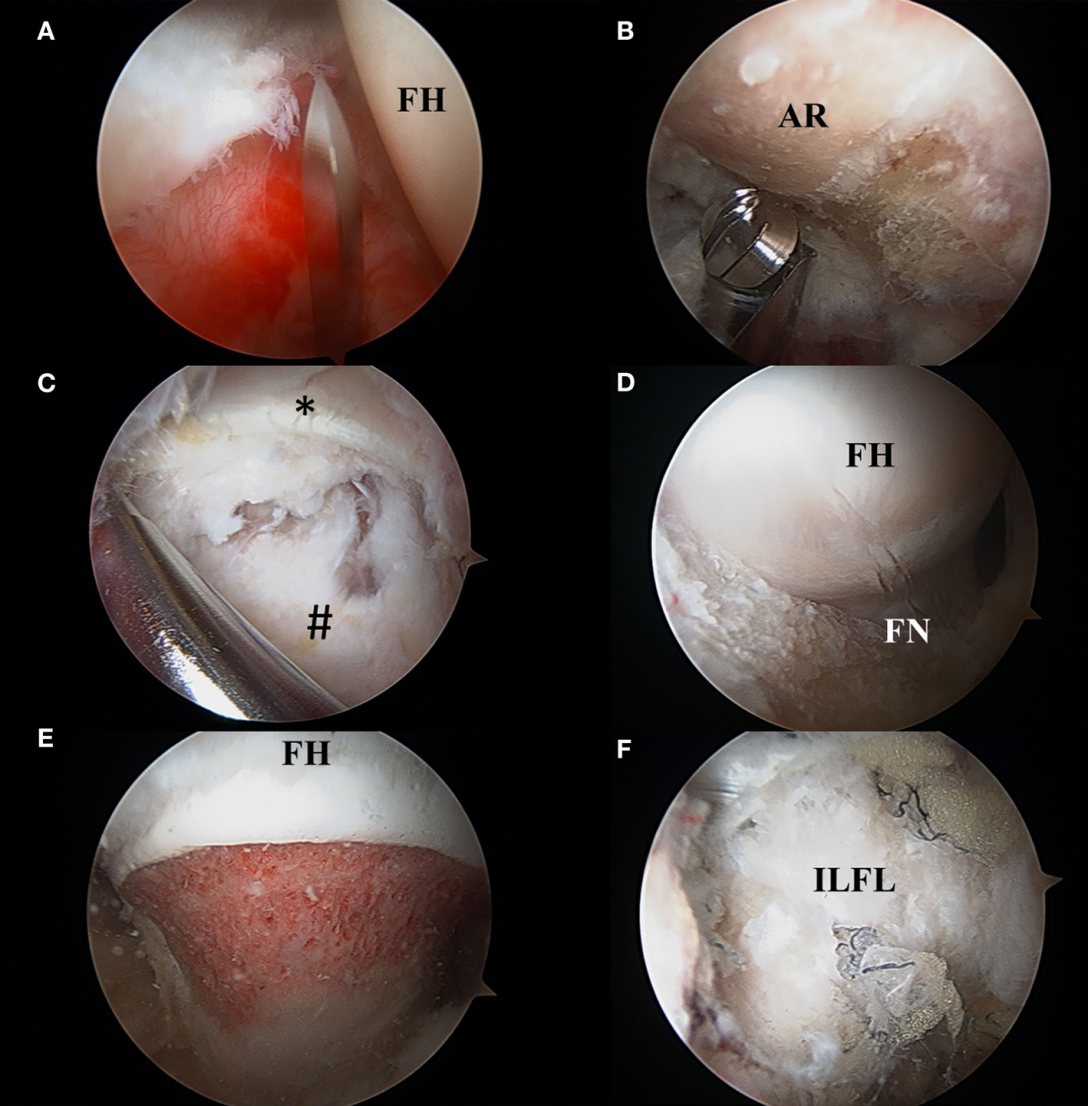
**(A)** Interportal capsulotomy as seen through the mid-anterior portal. The capsulotomy must begin at least 5 mm from the labrum to ensure repair and is in between 2 and 4 cm in length dependent on central compartment pathology. FH: femoral head. **(B)** Acetabular rim trimming. Viewing through the mid-anterior portal, pincer lesions are resected with an arthroscopic burr in the anterolateral working portal. AR, acetabular rim. **(C)** Viewing from the mid-anterior portal, the peripheral compartment is accessed through a T-capsulotomy. *Reflected head of the rectus femoris. ^#^Iliofemoral ligament. **(D)** T-capsulotomy extends down the femoral neck to expose the cam deformity. FH, femoral head; FN, femoral neck. **(E)** Completed femoral osteochondroplasty with resection of the cam lesion. FH, femoral head. **(F)** Appearance of capsule following complete repair of the T-capsulotomy. ILFL, repaired iliofemoral ligament.

Accurate portal placement is also important to ensure safety and appropriate visualization during hip arthroscopy in FAI (Figure 7). The AL portal is placed 1 cm anterior to the superior margin of the greater trochanter and pierces the gluteus medius before it enters the hip capsule, with the superior gluteal nerve traveling 4cm superior to portal insertion (35, 42). Additional portals involved with the central compartment include the anterior portal, located slightly lateral to the intersection of a horizontal line from the AL portal and a vertical line from the ASIS, and the PL portal, located 1 cm posterior and superior to the greater trochanter (37, 43). The anterior portal pierces the sartorius and rectus femoris before reaching the hip capsule, and branches of the LFCN are most at risk to injury (35). An interportal transverse capsulotomy between the anterior and AL portals is often created to increase visibility and mobility when working in the central compartment (Figure 8A) (44). The PL portal perforates the gluteus medius and minimus and enters the joint through the posterior edge of the lateral capsule (35). In a cadaveric study, Thorey et al. report central compartment visualization of the AL portal to be between 2:00 and 6:00, of the anterior portal to be 8:30 and 4:00, and of the PL to be 5:30 and 12:00 (45).

Many accessory portals have also been described and include the mid-anterior portal (MAP), proximal MAP (PMAP), proximal AL accessory (PALA), peritrochanteric space portal (PSP), and the distal AL accessory portal (DALA) (Figure 7) (42). After work in the central compartment is completed, traction is released, and the peripheral compartment is commonly accessed via an intra-capsular approach from the AL portal (43, 46). Alternately, for particularly complex cases in which access to the central compartment is difficult, the peripheral compartment can be entered primarily (47–49). As discussed previously, most patients with FAI have a mixed presentation with both pincer and cam deformities; therefore, access to both the central and peripheral compartments is generally required.

### Technique: Resection of Cam and Pincer Lesions

Procedures to correct the bony abnormalities in FAI include acetabular rim trimming for pincer lesions and femoral osteochondroplasty to correct cam deformities (Figures 8B–E). To access the pincer lesion, the labrum is usually taken down at the chondrolabral junction and reattached with suture anchors following acetabuloplasty (30, 41). Once the acetabular overcoverage has been identified, the lesion can be resected by trimming with an arthroscopic burr. One recent report describes a technique of labral preservation when addressing pincer lesions, in which the entire chondrolabral complex is lifted subperiosteally off the acetabular rim, and the pincer lesion is resected under fluoroscopic guidance (50). This technique has the advantage of preserving the chondrolabral transitional zone, which has been suggested as having suboptimal healing capabilities (51). Additionally, Redmond et al. recently reported identical 2-year outcome scores and revision rates in patients that underwent labral preservation compared to those that underwent takedown and reattachment for treatment of pincer lesions (52). Management of large pincer lesions can be particularly challenging as the acetabular overcoverage can block access to the central compartment through the AL portal (53). Pincer lesions can be exacerbated by additional hip deformities including acetabular retroversion, acetabular protrusion, and coxa vara/breva (53, 54). Large pincer lesions can be managed through a *capsulotomy first approach* where the hip is distracted following, rather than prior to, a capsulotomy. In this approach, the central compartment can be accessed via an inside–out technique in which the capsulotomy is performed from the peripheral compartment (53). Alternately, an *acetabuloplasty first approach*, in which the pincer lesion is first addressed in the peripheral compartment, until the central compartment can be accessed, can be utilized (53, 54). Notably, these approaches to large pincer lesions use primary peripheral compartment access before completing the operation in the central compartment.

Cam lesions are resected in the peripheral compartment, typically after work in the central compartment is completed. The hip is flexed to 30°, and traction is released to relax the anterior capsule. Work in the peripheral compartment is generally performed through the AL, MAP, and DALA portals (55, 56). In the setting of a cam lesion, the abnormal femoral neck offset is corrected via femoral osteochondroplasty, with adequate resection confirmed via a dynamic fluoroscopic examination (Figure 8E) (55). Adequate visualization in the peripheral compartment is critical, as inadequate cam resection remains a common reason for re-operation and inferior outcomes (57, 58). When working through the DALA portal, extension of the transverse capsulotomy from the femoral head/neck junction to the intertrochanteric line creates a T-type capsulotomy that affords increased visualization of cam lesions in the peripheral compartment (Figures 8C,D) (44, 56). The T-capsulotomy, while increasing arthroscopic visualization, potentially promotes increased femoral head translation within the acetabulum if left unrepaired (59, 60). In a case-control study of 64 patients, Frank et al. report significantly higher 6 months, 1 year, and 2.5 years hip outcome score-sports subscale (HOS-SS) for patients that had complete repair rather than partial repair of the T-capsulotomy (Figure 8F) (61). Conversely, Domb et al. found no differences between repaired and unrepaired capsulotomies when variables, such as age and preoperative cartilage damage, were controlled for (62). Capsular management in hip arthroscopy is controversial and remains an active topic of investigation.

The relative success of hip arthroscopy is often dependent on the adequacy of lesion resection; therefore, methods to improve both preoperative and intraoperative understanding of the bony anatomy are being actively investigated (58). In one recent study, Milone et al. report that 3D CT software reconstructions illustrated larger alpha angles when compared with 2D CT and Dunn lateral radiographs (63). Additionally, intraoperative fluoroscopy can be utilized by the arthroscopist to ensure adequate resection or cam lesions (64). When comparing six standardized intraoperative fluoroscopic views to a reconstructed preoperative CT, Ross et al. found that this method was able to successfully localize and ensure the appropriate amount of cam resection (65). Other forms of intraoperative imaging, such as image-based navigation and computer-aided navigation, have been used on an experimental basis to resect cam lesions but are not used in common practice (66, 67).

### Postoperative Management: Rehabilitation, Complications, and Re-Operation

Hip arthroscopy, with its minimally invasive approach, often offers a shorter recovery time than open hip preservation surgery. Return to activity can range from as short as 3 months for elite athletes to 9 months or longer for patients with poor preoperative muscle tone (33, 68). Rehabilitation may also be prolonged for patients with increased time between symptom onset and surgery (33). The overall rehabilitation process consists generally of four phases, and specific protocols are highly dependent on both the surgical procedure performed and surgeon preference (69). In our practice, following arthroscopic resection of cam and pincer lesions, *phase one* begins immediately postoperatively with limited foot flat or toe touch weight bearing for the first three weeks. If microfracture was performed for chondral defects, limited weight bearing is extended to last between 4 and 8 weeks (69). The goals of phase one are to protect the joint by avoiding inflammation and maintain appropriate passive range of hip motion (69–71). *Phase two* focuses on a return to non-compensatory gait with a focus on improving neuromusculature control and restoring full range of motion at the hip (71). *Phase three* emphasizes a return to preoperative levels of strength and conditioning and includes functional exercises designed to strengthen lower extremity and core musculature (70). *Phase four* concentrates on the return to preinjury sport and recreational activity level by working on maximizing plyometric strength, agility training, and sport specific exercises (70, 71). Specific physical therapy techniques tend to vary, with most protocols including CPM, soft tissue mobilization (STM), isometric stretching, and joint mobilization (69–72). While each phase generally lasts a minimum of 4 weeks, it is critical to ensure that the patient’s recovery guides rehabilitation and that any exacerbation in pain or limitation in activity is promptly addressed (69, 73).

Hip arthroscopy for FAI is not without risk, and while rare, reported complications are increasingly scrutinized. Recent literature reviews have found complication rates following hip arthroscopy to range from 1.5 to 7.5%, with most complications being minor and transient (33, 40, 74–77). Common minor complications include neuropraxias related (related to traction, perineal compression, or portal placement), iatrogenic chondral and labral damage, superficial infection, deep vein thrombosis, and HO (40, 76, 77). Comprising 37% of all complications, the most common minor complication is postoperative nerve injury related to either distraction or compression against the perineal post (77). One recent systematic review of 92 studies found a total nerve injury rate of 1.4% with 99% of these being temporary neuropraxias (76). HO following hip arthroscopy is generally rare, occurring in <1% of cases in two recent reviews, but reported rates have been as high as 44% (76–79). The low HO rates currently reported may be attributable to the use of prophylactic postoperative naproxen, which has been shown to dramatically decrease the rate of HO (78). Iatrogenic chondral and labral injuries were found to occur in up to 4.6% of cases; however, the clinical relevance of these injuries remains unclear (76, 80). Additionally, complication rate is dependent on surgeon experience, with one study reporting a statistically significant decrease in traction related complications after the surgeons’ first one hundred cases (81). In the largest study to date investigating 2-year outcomes following primarily hip arthroscopy for FAI, Gupta et al. report, out of a cohort of 595 consecutive surgeries, complications included 13 (2.1%) cases of postoperative neuropraxia, 14 cases of (2.35%) HO, three (0.5%) DVTs, five (0.84%) superficial wound infections, and one (0.17%) deep wound infection requiring irrigation and debridement (82).

Occurring in <1% of cases, major postoperative complications can be devastating and include deep infections, pulmonary emboli, skin damage, extra-articular extravasation requiring surgical decompression, vascular injury, avascular necrosis, femoral neck fractures, and frank dislocation (40, 76). Fluid extravasation inducing abdominal compartment syndrome can be a potentially life threatening complication (83, 84). It is recommended to minimize the intra-articular pressure when possible as well as to periodically check for hypotension, which can herald extravasation (40). Iatrogenic hip dislocation is another feared complication that has been related to capsulotomy without repair and excessive acetabular rim trimming (76, 85–89). Femoral neck fractures have also been recorded after femoral osteochondroplasty, often related to a premature return to full weight bearing (76, 77). Avascular necrosis of the femoral head has also been reported and may be caused following damage to lateral epiphyseal branches of the MFCA within the lateral synovial fold following femoral neck osteoplasty or t-type capsulotomy (74, 76, 77).

Revisions’ surgery following primary hip arthroscopy is rare, with recent studies reporting rates between 4.0 and 7.7% occurring on average 28 months following the primary surgery (57, 76, 77, 82). Harris et al. found that most re-operations used the open approach (70%) and were primarily for conversion to total hip arthroplasty. Revision arthroscopy is often reserved for loose body removal, lysis of adhesions, and most commonly, resection of residual cam or pincer lesions that were not adequately resected at the primary surgery (76, 77). Revision hip arthroscopies are generally successful, with significantly improved clinical outcomes, and a 5–14.6% re-operation rate (57, 90).

## Conclusion

While arthroscopic surgery has shown slightly superior short- and mid-term outcomes, it is not without risk, particularly in light of the steep learning curve to gain technical proficiency. Moreover, there are some instances where complex joint morphology, such as combined dysplasia and FAI, may preclude arthroscopy in favor of an open approach. Currently, there remains a paucity in both long-term outcomes as well as high-level randomized controlled trials comparing the open and arthroscopic approaches. However, four RCTs investigating this question are ongoing and may provide further clarification within the next few years (91). As with other surgeries, the approach taken to FAI must be individualized to reflect both patient anatomy and preference while at the same time accommodate surgeon comfort and technical skill.

## Conflict of Interest Statement

The authors declare that the research was conducted in the absence of any commercial or financial relationships that could be construed as a potential conflict of interest.
